# Imaging genetics in adult attention-deficit/hyperactivity disorder (ADHD): a way towards pathophysiological understanding?

**DOI:** 10.1186/2051-6673-1-6

**Published:** 2014-05-12

**Authors:** Thomas Dresler, Beatrix Barth, Thomas Ethofer, Klaus-Peter Lesch, Ann-Christine Ehlis, Andreas J Fallgatter

**Affiliations:** Department of Psychiatry and Psychotherapy, University of Tübingen, Tübingen, Germany; LEAD Graduate School, University of Tübingen, Tübingen, Germany; Graduate School of Neural and Behavioral Sciences, University of Tübingen, Tübingen, Germany; Division of Molecular Psychiatry, Department of Psychiatry, Psychosomatics and Psychotherapy, University of Würzburg, Würzburg, Germany; CIN, Center of Integrative Neuroscience, Excellence Cluster, University of Tübingen, Tübingen, Germany

## Abstract

Attention-deficit/hyperactivity disorder (ADHD) is a common, early-onset and enduring developmental disorder whose underlying etiological and neurobiological processes are the current focus of major research. Research strategies have made considerable effort in elucidating the complex genetic architecture of ADHD and indicate various pathways from genotype to phenotype. Understanding ADHD as a neuropsychiatric disorder enabled to investigate markers of neural activity as endophenotypes to better explain the link from gene to symptomatology (the so-called imaging genetics approach). Overcoming the originally rather restrictive requirements for an endophenotype, imaging genetics studies are supposed to offer a much more flexible and hypothesis-driven approach towards the etiology of ADHD. Although 1) ADHD often persists into adulthood, thus remaining a prevalent disorder, and 2) imaging genetics provides a promising research approach, a review on imaging genetics in adult ADHD – as available for childhood ADHD (Durston 2010) – is lacking. In this review, therefore, findings from the few available imaging genetics studies in adult ADHD will be summarized and complemented by relevant findings from healthy controls and children with ADHD that are considered important for the adult ADHD imaging genetics approach. The studies will be reviewed regarding implications for basic research and possible practical applications. Imaging genetics studies in adult ADHD have the potential to further clarify pathophysiological pathways and mechanisms, to derive new testable hypotheses, to investigate genetic interaction effects and to partly influence practical applications. In combination with other research strategies, they can incrementally foster the understanding of relevant processes in a more comprehensive way. Current limitations comprise the incapability to discover new genes, a high genetic load in patients potentially obscuring the effect of single candidate genes, the mostly unknown heritability of the endophenotype and the heterogeneous manifestation of ADHD.

## Introduction

Attention-deficit/hyperactivity disorder (ADHD) represents a common, early-onset and enduring neurodevelopmental disorder with a worldwide-pooled prevalence of 5.3% in childhood and adolescence [1]. In adulthood, ADHD-related symptoms may still cause impairments and its pooled prevalence still amounts to 2.5% [2, 3], so it cannot be regarded a mere transitional childhood disorder. Both childhood and adult ADHD have a large economic impact on society [4] and have been in a major focus of research in the last decades (see [5, 6]).

Research strategies have made great efforts in elucidating the complex genetic architecture of childhood and adult ADHD [7, 8]; association and linkage studies have revealed relationships of specific candidate genes with ADHD and with disorder-specific symptom scales, pointing towards pathophysiologically relevant genetic influences. The understanding of ADHD as a neuropsychiatric disorder (e.g., [9]) further enabled the investigation of the influence of specific genetic variations on markers of neural activity to better explain the link from gene to disorder and symptoms, respectively [10]. In such an *imaging genetics* approach neuroimaging data are treated as endophenotypes [10, 11], which are assumed to be more closely related to the underlying cellular and pathophysiological processes than the symptomatic behavior or the categorical diagnoses themselves. The pathway from gene to behavior is complex and characterized by different steps (see Figure one in [12]): variation in gene expression results in differential availability of proteins, which are necessary for specific structural and functional properties of neurons that make up neural units and circuits. The response of these circuits can be measured using various neuroimaging techniques such as functional magnetic resonance imaging (fMRI), positron emission tomography (PET), functional near-infrared spectroscopy (NIRS), magnetoencephalography (MEG) and electroencephalography (EEG). The first imaging genetics studies were published around the millennium (e.g., [13]) when imaging methods became more broadly available. Since then, the number of publications on imaging genetics has increased considerably and studies where applied to various neuropsychiatric disorders and related (endo)phenotypes.

Although the endophenotype concept for neuropsychiatric disorders [14] was rather restrictive and axiomatic when being first introduced (e.g., [11]), in recent years notions for a reconceptualization have been proposed. For example, Kendler and Neale [15] stress the necessity of differentiating between mediational (i.e. the endophenotype mediates the relation from gene to disorder) and liability index models (i.e. the endophenotype is risk-indicating and may only be an epiphenomenon), thus broadening the original framework. Furthermore, endophenotypes and thus imaging data could reflect environmental influences; it is also feasible to assume that some genetic influences only affect the endophenotype while others only affect the clinical symptoms [15]. Taken together, this illustrates that current concepts of (neuroimaging) endophenotypes may provide new insights into the pathophysiological processes and mechanisms as several influencing factors are considered and as the originally rather restrictive requirements for an endophenotype do not have to be fulfilled. This allows a much more flexible and hypothesis-driven approach towards the etiology of ADHD. Yet, imaging genetics in ADHD is still in its infancy and studies are scarce [16].

Although 1) ADHD often persists into adulthood, thus remaining a prevalent disorder, and 2) imaging genetics provides a promising research approach, a review on imaging genetics in adult ADHD – as available for childhood ADHD [17] – is lacking. In the present review, we will first summarize the findings from the few available imaging genetics studies in adult ADHD and subsequently discuss the various potentials and challenges in pathophysiological research and clinical implications assuming a more flexible understanding of endophenotypes [15].

## Review

Literature searches using available data bases (pubmed, google scholar) were conducted to find imaging genetics studies in adult samples of ADHD (till May 2013). Eight studies (4 fMRI, 4 EEG) were then classified into two categories, i.e. cognitive and affective-motivational approaches, depending on whether they applied cognitive or emotional-motivational paradigms.^a^ Although this distinction is arbitrary and may not always be unambiguous, it should help to better organize the available data. Table 1 summarizes the reviewed studies and Table 2 provides information about the effects the gene variants have.

**Table 1:** **Studies on imaging genetics in adult ADHD**

Authors	Participants	Paradigm	Gene variant	Imaging	Imaging genetics results
Baehne et al. [21]	124 patients	Go/NoGo task (CPT)	TPH2 (G-allele polymorphism in rs4570625, T-allele polymorphism in rs11178997)	EEG (NGA)	Reduced NGA in risk allele carriers in ADHD and healthy controls
	84 controls				
Dresler et al. [24]	161 patients	Go/NoGo task (CPT)	SLC6A3 (3′ UTR VNTR)	EEG (NGA)	Reduced NGA in 9-repeat allele carriers in the patients, no influence in healthy controls
	109 controls				
Fallgatter et al. [29]	216 patients	Go/NoGo task (CPT)	LPHN3	EEG (NGA)	Reduced NGA in the LPHN3 high risk group
Heinzel et al. [25]	181 patients	Go/NoGo task (CPT)	COMT (Val158Met), DRD4 (exon 3 VNTR)	EEG (NGA)	Significant DRD4 × COMT interaction on NGA (DRD4 no7R: inverted u-shape with increasing COMT-dependent DA levels, DRD4 7R: u-shape), no gene main effects, no interaction with group
	114 controls				
Brown et al. [23]	52 patients	Working memory (n-back)	SLC6A3 (3′ UTR VNTR)	fMRI	Marginal reduced left mePFC signal in 9-repeat allele carriers in patients and controls, marginal genotype-by-diagnosis interaction in the SMA/dACC (increased activation in 10-repeat allele homozygous patients vs. controls)
	38 controls				
Brown et al. [30]	42 patients	Multi-source interference task	SLC6A3 (3′ UTR VNTR)	fMRI	Hypoactivation in 9-repeat allele homozygous patients in the left dACC
Hoogman et al. [32]	63 patients	Delay discounting task	NOS1 exon 1f-VNTR	fMRI	SS-allele carriers demonstrate higher ventral striatum activity in patients
	41 controls				
Hoogman et al. [34]	87 patients	Delay discounting task	SLC6A3 (3′UTR VNTR/intron 8 VNTR haplotype)	fMRI	No significant effects of DAT1 haplotype on striatal activity
	77 controls				

CPT, continuous performance test; DA, dopamine; dACC, dorsal anterior cingulate cortex; EEG, electroencephalography; fMRI, functional magnetic resonance imaging; mePFC, medial prefrontal cortex; NGA, NoGo-anteriorisation; SMA, supplementary motor area; UTR, untranslated region; VNTR, variable number of tandem repeats.

**Table 2:** **Effects of gene variants**

Gene variant	Gene product	Gene product function	Influence of variant	Primary brain regions of action	Physiologic effects
**Dopamine system**					
COMT (Val158Met)	Catechol-o-methyltransferase	Degradation of catecholamines	Met variant- carriers have reduced COMT activity	Prefrontal Cortex	Differential dopaminergic signaling influences PFC function (COMT-genotype model), may explain different drug effects [58]
DRD4 (exon 3 VNTR)	Dopamine receptor D4	Dopaminergic transmission	7R-carriers have reduced DRD4 function	Prefrontal Cortex	Variation in dopaminergic signaling influences PFC function, interaction with other genotypes [25]
SLC6A3 (3′UTR VNTR); SLC6A3 (3′UTR VNTR/intron 8 VNTR haplotype)	Dopamine transporter	Reuptake of dopamine	9R- vs. 10R-carriers have reduced or increased DAT availability (inconsistent findings)	Striatum	Variability of striatal dopamine Transporter availability influences PFC function directly or indirectly via cortico-striatal pathways [24]
**Serotonin system**					
TPH2 (e.g. rs4570625, rs11178997)	Tryptophan hydroxylase 2	Synthesis of serotonin	Influences transcriptional activity	Raphe nuclei, with ubiquitous action of serotonin	Differential activity of the cortico-limbic circuit
**Others**					
NOS1 exon 1f-VNTR	Neuronal nitric oxide (NO) synthase	Synthesis of neuronal NO	Allelic variation in reporter gene expression	Striatum	NO function influences dopamine signaling [32]
LPHN3 (ADHD risk haplotype)	Latrophilin	Adhesion G-protein coupled receptor (?)	Decreased NAA/Cr ratio in risk haplotype carriers	Amygdala, caudate nucleus, cerebellum, and cerebral cortex	Possibly influences dopamine-glutamatergic system interaction

COMT, Catechol-o-methyltransferase; DAT, dopamine transporter; NAA/Cr, N-acetyl aspartate/creatine; NO, nitric oxide; PFC, prefrontal cortex; UTR, untranslated region; VNTR, variable number of tandem repeats.

### Cognitive paradigms

Patients with childhood and adult ADHD show impairments in different cognitive domains [18, 19] which represent a core feature in ADHD-related psychopathology (e.g., [5]). The available imaging genetics studies have implemented different cognitive paradigms.

#### Go/NoGo paradigms

Continuous performance tasks (CPT, see [20]) involve the combination of response execution (in Go trials) and response inhibition processes (in NoGo trials) and thus higher-order motor control and attention. In a series of electroencephalographic (EEG) studies, our research group has applied an OX version of the CPT in a large sample of adult ADHD patients investigating the influence of several gene variants on behavioural and neural parameters. In these studies, we derived a topographical event-related potential (ERP) parameter, the so-called NoGo anteriorization (NGA), which has been proposed to reflect mechanisms of prefrontal response control. Basically, the individual NGA is calculated as the difference between the mean area centroids of the P300 field maps for the Go and NoGo condition. In one study [21], for two ADHD-associated gene variants within the tryptophan hydroxylase 2 (TPH2) gene (i.e. rs4570625, rs11178997) the risk alleles were associated with reduced NGA in both patients and controls. On the behavioral level, the expected pattern of lower performance in ADHD was found. In a subsequent study, the dopamine transporter (DAT, SLC6A3) gene was investigated. It had been found that in adult ADHD – contrary to childhood ADHD – the 9-repeat allelic variant of SLC6A3 represented the risk allele [22, 23]. Therefore, we hypothesized for our adult sample that the 9-repeat allele should be associated with decreased prefrontal function. In line with this hypothesis we found that – only in patients – the 9-repeat allele was associated with a reduced NGA, while there was no significant effect in the control group [24]. Recently, we were able to show that – across ADHD patients and controls – there was no main effect of two common gene variations (i.e. catecholamine-O-methyl transferase [COMT] val158met polymorphism; dopamine receptor D4, DRD4 variable number of tandem repeats [VNTR]) on behavioral performance or the NGA, but an interaction between the gene variants on the investigated parameters [25]. Such gene-by-gene interaction effects may offer new insights into the interplay of various genes at the physiological level. During the last years, a rather unexpected genetic candidate came into focus of ADHD research, i.e. common haplotype of the latrophilin 3 (LPHN3) gene that has been found to be associated with the disorder in pedigrees and population-based studies [26, 27]. It has also been shown to modulate dopaminergic neuron formation and locomotor activity/impulsivity in zebrafish, whereby effects can be influenced by methylphenidate [28]. Given this, we also found that having two risk alleles of the LPHN3 gene was associated with a decreased NGA [29].

#### Working memory paradigms

In these tasks, subjects are required to maintain or manipulate information in memory to fulfil specific experimental conditions. In an fMRI study, Brown et al. [23] applied a sequential letter visual n-back task and investigated whether task-specific activation contrasting 2- vs. 0-back was influenced by group or DAT genotype. While no differences emerged on the behavioral level, a marginal association of the DAT 9-allele with increased task-related suppression in the left medial PFC and a marginal genotype × diagnosis interaction in the dorsal anterior cingulate cortex (dACC) was found. According to the authors, the finding suggests that the task-related suppression in the default mode network (DMN) might act as an intermediate phenotype between DAT1 and ADHD.

#### Interference paradigms

These tasks require subjects to ignore specific (task-irrelevant) information to perform the experimental conditions correctly. In an ADHD group, Brown et al. [30] investigated mediating effects of the DAT1 gene on dACC function in a multi-source interference task (MSIT) and found less activation in homozygous 10- as compared to 9-repeat allele carriers when confronted with response-incongruent interfering stimuli.

### Affective-motivational paradigms

In the emotional domain, ADHD patients also display various disturbances [31]; however, these have so far rarely been investigated in imaging genetics studies.

#### Reward paradigms

Hoogman et al. [32] applied a delay discounting task in which subjects had to choose between hypothetical immediate or delayed rewards. fMRI data indicated a generally increased ventral striatal activity in controls vs. ADHD patients; however, across both groups homozygous carriers of the short allele of the nitric oxide synthase (NOS1) gene (for more information see [33]) exhibited increased activity which – in the patients – was accompanied by higher impulsivity. Therefore, the authors assume the NOS1 influence on ADHD to be mediated by its effect on impulsivity. In another study, Hoogman et al. [34] investigated the influence of a DAT haplotype on ventral striatum activation during reward anticipation. Here, besides the *a priori* expected increased ventral striatal activity in controls vs. ADHD patients, no effect of the haplotype could be discerned.

Paloyelis et al. [35] argued against a deficit in anticipation-related neural activation in the ventral striatum. Instead they suggested dysfunctional processing of reward outcomes in adolescents with combined ADHD subtype (ADHS-CT). They further highlighted the role of genes influencing dopamine signaling in reward-processing as a DAT haplotype was found to modulate striatal responsivity during incentive-predicting stimuli. DAT 10/6-repeat allele homozygosity was associated with decreased reward-related anticipatory striatum activity in the ADHD-CT on the one hand, but increased neural activation in healthy controls on the other hand. In how far these findings also apply to adults has yet to be investigated.

### Research on other endophenotypes and genes

As the number of available imaging genetics studies in adult ADHD is limited so far, we will shortly refer to exemplary findings in healthy subjects and children. Especially studies on emotional processing are promising. Behavioral studies reveal that ADHD children are less accurate in classifying emotional facial expressions [36–38]. On some measures they even tend to perform worse than autistic children [39] and seem to be unaware of their compromised social cognition [40]. These perceptual deficits can interfere with executive functions [41] and are not restricted to facial expressions, but occur in a similar way for vocal emotions [36, 37]. Behavioral studies in adult ADHD indicate that deficits in judgment of facial expressions persist to adulthood [31]. The neural correlates of this disturbed perception of emotional signals as well as their genetic origins, however, are largely unknown and warrant further investigation.

Neuroimaging studies in healthy participants provided evidence for distinct pathways for explicit versus implicit processing of emotions in faces [42] and voices [43]. Studies on explicit processing of emotions in childhood ADHD [44] revealed a hyperactivation of the amygdala during such paradigms. This enhanced activation of the amygdala in ADHD patients is in line with structural findings [45] as well as with a reduction of dopamine receptors [46] in this brain area. Genetic imaging studies in healthy subjects demonstrated that brain activation during emotional processing can be strongly influenced by the genotype of key enzymes of dopaminergic pathways including DRD4 [47] and DAT [48]. These genes have been associated with an increased risk for ADHD (for a review see [7, 27]). In particular, the high-activity genotype of COMT is a strong candidate for mediating altered emotional perception in ADHD since it has been shown to be linked to antisocial behavior in this patient group [49]. Similarly, recently described genes such as the brain-expressed GTP-binding RAS-like 2 gene DIRAS2 [50] and LPHN3 [27, 49] have also been shown to be associated with personality traits that predispose to socially disruptive behavior. Further candidate genes derived from imaging genetics studies in healthy adults that might impact perception of emotional stimuli in ADHD include the TPH2, which has been shown to modulate amygdala activity to emotional stimuli (rs4570625 G-allele polymorphism, [51]), as well as Stathmin 1 (STMN1), a neuronal growth associated protein which is highly expressed in the amygdala that modulates the P300 in an emotional Stroop task (rs182455 C-allele, [52]). Considering overlapping symptoms with borderline personality disorder (BPD) [53], studying mechanisms related to emotional dysregulation may clarify shared pathways.

Apart from dysfunctional emotional processes, other endophenotypes need to be considered in further studies, e.g., neuropsychological deficits, such as measures of attention, executive function and processing speed [54]. Furthermore, also other genes in the dopaminergic, serotonergic, noradrenergic and neurotrophic system need to be considered [7].

## Discussion

So far, imaging genetics studies in adult ADHD are – compared to other lines of ADHD research – rather scarce and research is still at its beginning. Nonetheless, the available findings provide an outlook on the potential chances of this research approach which – in combination with complementary approaches – may help to increase our knowledge about the pathophysiological pathways in adult ADHD. Yet, several limitations need to be considered as well (see below).

### Promises

The imaging genetics approach provides an improved insight into possible, important pathophysiological pathways and it allows deriving testable hypotheses that promote a better understanding of the neurobiology of normal and pathophysiologically altered behavior. Studies reporting that gene variants influence neural markers independently of diagnostic status (e.g., [21, 23, 25, 32]) hint at common neural pathways in patients and controls. Such influences are in line with a more continuous model of ADHD phenotypes going beyond simple categorical disorders. Assuming such a continuously distributed trait in the population [55, 56] – with ADHD being defined by an arbitrary threshold on this trait – may allow to estimate the individual risk for passing this threshold taking into account several single candidate genes. Studies only showing associations in ADHD patients, but not in healthy controls (e.g., [24]), hint at more disorder-specific pathways that may influence brain activity – and therefore clinically relevant behavior – independently of shared processes. Here, the presence of specific gene variants may indicate specific (neuropsychological) symptom dimensions, e.g., neural impairment in executive functions.

Candidate genes that have been replicated empirically in large-scale association studies (e.g., DAT) can be tested regarding their functional impact in a hypothesis-driven approach. These genetic findings indicate an involvement of specific psychopathological pathways which can be further investigated in imaging genetics studies: For example, the association of the DAT 9-repeat allele with adult ADHD led to our hypothesis that this allele – which influences the frontal-striatal loop – may be associated with a concurrent less efficient prefrontal functioning in our adult ADHD sample [24].

Imaging genetics has the potential to be used in a translational manner, thus allowing basic findings to influence practical applications. If specific gene variants are highly associated with altered brain activation in specific cognitive or emotional domains, a more tailored pharmacological or psychotherapeutic intervention can be chosen, thus offering new treatment targets [57]. Mattay et al. [58] found that COMT val/val carriers (i.e. carriers of the high enzyme activity variant putatively showing suboptimal prefrontal function at baseline) displayed more efficient prefrontal function in a working memory task after an amphetamine challenge, whereas in met/met carriers counterproductive effects were observed at high working memory loads. Studying schizophrenic patients, Ehlis et al. [59] found the val/val allele to be associated with lower performance and a reduced NGA; furthermore, they showed that patients responded better to typical or atypical antipsychotics depending on their NGA values before treatment [60]. Using and combining such knowledge may help to more carefully adjust treatment regimes and to reduce side effects.

Complex interactive effects of several genes with individually small impact [61] have been suggested to underlie ADHD. The increasing number of large-scale studies will enable to investigate putative gene-by-gene interactions and their influence on ADHD-related behavior and neurophysiology (e.g., [25]). Beyond, also environmental factors contribute to ADHD; however, little is known about precise gene-by-environment interactions on the neural level. Therefore, gene-by-environment interactions could be a further target in such studies augmenting the data from behavioral studies and helping to clarify existing inconsistencies [62]. Integrating imaging genetics to investigate these interactions (for an overview see [63]) and distinct endophenotypes can lead to more precise statements concerning direct versus indirect effects of genes or environment on ADHD.

Imaging genetics is one element that in combination with other – also distant – research strategies (e.g., *in vivo* and *in vitro* investigations, animal models, combined PET-fMRI measurements) helps to identify important pathophysiological mechanisms. For example, the research on the risk haplotype in the LPHN3 gene originated from pedigree linkage studies in a Columbian genetic isolate [26] and was subsequently complemented by association samples in Caucasian samples [27, 64]. This convinced us to stratify our ADHD sample regarding this haplotype and we indeed found a reduced prefrontal functioning in the subgroup with two risk alleles [29]. At the same time, a study investigating the ortholog lphn3.1 in the zebrafish revealed that loss of its function resulted in misplaced diencephalic dopaminergic neurons and hyperactive and impulsive swimming behaviour; furthermore, this could be reversed by the common ADHD drugs methylphenidate and atomoxetine [28]. These findings fit with disturbances in the development of the dopaminergic system being influenced by this protein/receptor complex whose actual function is not well understood. As LPHN3 is not a direct key player in the dopaminergic system, its functional impact has to be further investigated. Here, imaging genetics may help to identify functional processes and underlying brain networks that are affected by gene variants and might link LPHN3 (albeit indirectly) to dopaminergic pathways.

### Current limitations of the reviewed studies

Up to now, most studies in imaging genetics, especially in clinical research, lack large sample sizes. Several factors may contribute to this: e.g., patient recruitment problems or financial issues. Moreover, no longitudinal studies investigating the impact of gene variants across a specific time-span are available. Particularly, the different association of DAT polymorphism with ADHD in childhood vs. adulthood should be further elucidated by longitudinal research.

Although these candidate gene studies provide important information in the context of a clear functional hypothesis, they are unable to discover new genes or their combinations [65]. A genome-wide approach could, but is not feasible in imaging genetics. Furthermore, only investigating single genes might be a restricted perspective on the complexity, as larger influences of non-candidate genes could be missed.

The imaging genetics approach might be problematic, as patients are prone to disorder-related confounds and hence carry a high genetic load which also includes low-frequency genes (see Box 7 in [66]). This may obscure the effect of single common candidate genes which are the main target of the articles. The investigation of ADHD genes in larger healthy samples could help to assess and avoid these confounds.

Often, the question remains whether the applied imaging paradigms are in fact heritable. To date, a few studies already indicate heritability of the brain activation during working memory [67, 68] and inhibition [69] which supports the assumption of a heritable endophenotype; however, for most of the paradigms heritability has not been tested.

As dopaminergic candidate genes have been extensively investigated in molecular genetics studies [7, 70], imaging genetics studies should more specifically target these to further elucidate intermediate neurobiological processes.

ADHD shows a considerable overlap in clinical symptoms (e.g., emotional dysregulation) with BPD. High genetic correlations for these disorders support the hypothesis that ADHD in childhood might even be a precursor of BPD in adulthood [71]. Thus, future imaging genetics studies should include patient samples of both disorders to clarify whether identified endophenotypes are specific or reflect a common pathway of these disorders that result in impaired emotion regulation.”

Another aspect which is often not considered in current research literature on ADHD is its rather heterogeneous manifestation. Refining research in this respect might reduce contradictory results and simultaneously enhance comparability between studies. Also in this context, imaging genetics could be one approach to define ADHD subtypes that are more strongly related to differences in underlying neurobiology than current “clinical” diagnostic classifications. Some studies focusing on DSM-IV subtypes (inattentive, hyperactive, combined type) did not provide any significant effects [72, 73], while others revealed age-specific and persistent as well as cross-subtype and subtype-specific gene influences [74]. Such findings highlight the need to control for factors such as age or comorbid symptoms in imaging genetics studies. Beyond that, the so far scarce evidence of a genetic basis for DSM-IV subtypes needs to be further substantiated. Alternatively, besides diagnostic subtypes, further neuropsychologically based models could be taken into account to cope with the heterogeneity in ADHD. For example, the dual pathway model [75], subsequently refined to a triple pathway model [76], suggests ADHD-CT to be either a motivational style or a dysregulation of inhibition which in turn are suggested to be based on different pathways in the dopamine system. Even though current models still differ in many ways and could be further corroborated, they share the notion of ADHD as a heterogeneous disorder including different subtypes based on alterations in distinct neural pathways. This common view should be considered when focusing on gene moderating or mediating effects on brain functioning in ADHD with the longer-term objective to diminish inconclusive or contradictory results.

### Limitations of the review

The review is based on only a few studies which prevents drawing strong unequivocal conclusions. These studies are also rather diverse with regards to paradigm, endophenoype measurement and investigated candidate genes.

## Conclusion

Taken together, current literature on the genetics of ADHD is not conclusive. Here, imaging genetic studies could come into play and lead to new insights regarding the complex and diverse pathways from the level of genetic vulnerability to overt clinical symptoms. Also, presently, the reported research findings are not directly applicable in clinical practice for adult ADHD (diagnostics, treatment, therapy) which is an aspiration for the near future. As in 30% of ADHD patients stimulant treatments do not lead to symptom improvements [77], further research on pharmacogenetics is required. Longterm-studies integrating neuropsychological and neuroimaging methods may advance our comprehension of underlying mechanisms in drug action. Up till now, only one study has combined functional near-infrared spectroscopy imaging and possibly moderating genotypes in a treatment study using methylphenidate [78]. So, in research on effective and long-term stable treatment, different ADHD subtypes should be taken into account using imaging genetics.

## Endnote

^a^One study [78] was not integrated as both adult and adolescent ADHD patients were investigated, but not analyzed separately.
